# Analyses of Contact Networks of Community Dogs on a University Campus in Nakhon Pathom, Thailand

**DOI:** 10.3390/vetsci8120299

**Published:** 2021-11-30

**Authors:** Tipsarp Kittisiam, Waraphon Phimpraphai, Suwicha Kasemsuwan, Krishna Kumar Thakur

**Affiliations:** 1 Department of Veterinary Public Health, Faculty of Veterinary Medicine, Kasetsart University, Kamphaeng Saen, Nakhon Pathom 73140, Thailand; fvetswk@ku.ac.th; 2 Department of Health Management, Atlantic Veterinary College, University of Prince Edward Island, Charlottetown, PE C1A 4P3, Canada; kthakur@upei.ca

**Keywords:** free-roaming dogs, contact network, network analysis

## Abstract

Free-roaming dogs have been identified as an important reservoir of rabies in many countries including Thailand. There is a need for novel insights to improve current rabies control strategies in these countries. Network analysis is commonly used to study the interactions between individuals or organizations and has been applied in preventive veterinary medicine. However, contact networks of domestic free-roaming dogs are mostly unexplored. The objective of this study was to explore the contact network of free-roaming dogs residing on a university campus. Three one-mode networks were created using co-appearances of dogs as edges. A two-mode network was created by associating the dog with the pre-defined area it was seen in. The average number of contacts a dog had was 6.74. The normalized degree for the weekend network was significantly higher compared to the weekday network. All one-mode networks displayed small-world network characteristics. Most dogs were observed in only one area. The average number of dogs which shared an area was 8.67. In this study, we demonstrated the potential of observational methods to create networks of contacts. The network information acquired can be further used in network modeling and designing targeted disease control programs.

## 1. Introduction

Free-roaming dogs include all unconfined dogs with varying relationships with humans. They can be grouped by their ownership status into three loose categories: owned dogs, semi-owned (which means the owner of the dog is not clearly defined, but the dog is associated with at least one human or household) or unowned dogs (sometimes referred to as feral dogs or stray dogs) [1]. Semi-owned dogs are also known as community dogs since they are related to multiple households in the community [2,3]. However, it is not always practical to correctly discern a dog from one subcategory to another without appropriate information. Free-roaming dogs can be found in multiple countries across Asia, including Indonesia (especially in Bali), the Philippines, India, Sri Lanka and Thailand [4]. In 2016, the Department of Livestock Development (DLD), Thailand, reported a total of 7.4 million dogs (6.6 million owned and over 700,000 unowned dogs). Dogs are commonly raised as free-ranging [5] and unowned dogs are often fed by people in the community [6]. Thus, free-roaming dogs can be found commonly in Thailand.

The potential public health and ecological health impacts from free-roaming dogs has led to developing various measures to control their population such as a capture-neuter-release program. The presence of free-roaming dogs living near wildlife conservation areas introduces a concern for the ecological impact of dogs as an invasive alien species [7]. With regards to their importance in public health, free-roaming dogs have been identified as a reservoir for rabies in many endemic countries [8]. Rabies is a lethal zoonotic disease. The World Health Organization (WHO) has asserted the importance of controlling rabies in dogs, especially in countries with free-roaming dogs, in order to eliminate human rabies [9]. Rabies is endemic in Thailand. Despite the collaborative efforts between the municipalities and the DLD to control the disease, there are still reports of positive cases detected through passive surveillance which suggest potential outbreaks [10]. Suboptimal vaccine coverage has been identified as one of the major obstacles in controlling rabies in Thailand [11]. Adjunct interventions such as orally-administered rabies vaccinations are being developed to increase the total vaccine coverage [12]. Strategically vaccinating dogs with higher than average contacts in addition to increasing efforts to vaccinate the free-roaming dog population, could be an effective strategy in controlling rabies. Hence, leading to the sufficient control of rabies spread in free-roaming dogs and preventing the spillover to humans. This has encouraged the researchers to develop a method to help identify high priority targets for vaccination.

Network analysis is a commonly used method to study the relationships between individuals. It has been utilized to study the contact networks of humans [13,14] and various animals, including lizards [15], dolphins [16] and non-human primates [17]. Furthermore, various networks of livestock movements such as cattle [18,19] and swine [20,21,22] have been studied in the interest of controlling disease spread. Network analysis is used in veterinary epidemiology to explain and model the relationship (such as contacts) between animals or group of animals based on movement, co-occurrence, proximity, etc., and the important role such contacts play in disease spread within and between groups [23,24,25].

Few studies describing contact networks of owned free-roaming dogs have been conducted. A study in Cheshire, UK [26], demonstrated the high level of potential contacts which could occur from dogs that share walking routes using a two-mode network. The observed network was highly connected and had small-world network characteristics that could support the fast spread of an infectious disease in the network [26]. Dog-to-dog networks were studied in Torres Strait, Australia [27] and in a separate study dog-to-dog networks in four countries (Chad, Guatemala, Indonesia and Uganda), were compared [28]. Both these studies also identified the small-world structure of the networks which could lead to rapid spread of disease. Spatial proximity was identified as an influential factor in the centrality of a dog in both studies [27,28]. Network-based simulation of rabies spread utilizing the network parameters [29,30] emphasize the role of network analysis in understanding how a disease can potentially spread in these populations and identify individuals or groups that can be targeted for surveillance and disease control. To the best of our knowledge, there has not been a study conducted on unowned or semi-owned dogs in Thailand. The objective of this study is to describe the contact patterns between dogs and the areas which they frequent on a university campus in Thailand by using network analysis. Additionally, the study aims to describe the network between the free-roaming dogs observed on the campus, as well as to characterize the network properties of both the dog-to-dog network and dog-to-area network to identify key nodes that have a potential for disease spread.

## 2. Materials and Methods

### 2.1. Study Site

**Figure 1:**
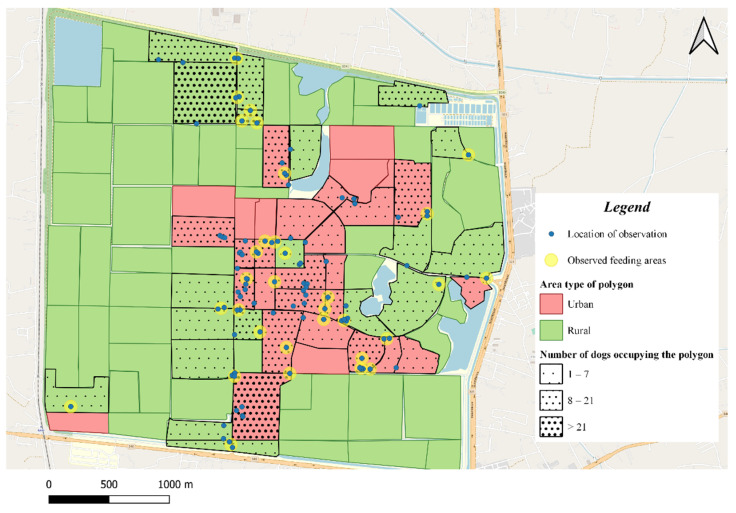
Map of Kasetsart University, Kamphaeng Saen Campus in 2020. The map of Kasetsart University, Kamphaeng Saen Campus was made using OpenStreetMap. The areas are defined as urban (pink) or rural (green), according to the level of human activity and the proportion of buildings in the polygon. The number of dogs occupying the polygon is depicted with the density of the black spots filled in the polygon. The locations of dogs observed in the study are displayed as blue triangular symbols on the map.

This study was conducted at Kasetsart University, Kamphaeng Saen Campus, which is located in Nakhon Pathom Province, in the Central Region of Thailand. The campus area is estimated to be over 3000 acres, most of which are agricultural fields. The campus has around 2000 staff and 14,600 students [31]. Free-roaming dogs are commonly found on campus. In a previous study [32], the campus area was divided into 96 polygons using roads and natural boundaries such as lakes in order to estimate the population of free-roaming dogs within the campus. For the purpose of this study, the 96 polygons were categorized into urban and rural types using two main criteria: assumed human activity and occupancy of human-made structures (not including plantations) (Figure 1). Additional information regarding the categorization of urban and rural types can be found in Appendix A.

In the most recent survey, at least 584 dogs were estimated to live on campus in 2019 [32]. The surveyed population only included community dogs or semi-owned dogs. Dogs in the previous study are routinely fed by staff of the university on weekdays which suggests that working hours could affect the grouping behavior of the dogs.

### 2.2. Data Collection

Visual observations were conducted to record dog sightings and their contacts. They were conducted by one observer (TK). Caretakers of community dogs on campus regularly feed the dogs before (6–7 a.m.) or after work (4–6 p.m.). In order to increase the chances of capturing the largest number of dogs during a cross-sectional observation, the planned observations were conducted between these specific times. During the sighting of a dog, a photograph was taken by a digital camera or a mobile phone. The dog’s overall physical appearance, such as the coat, coat color and obvious visible markings, were recorded along with its age, sex and castration status when possible. Neuter status was assumed from the absence of testes in male dogs. However, for female dogs, ovariohysterectomy does not leave a noticeable scar. Therefore, they are assumed to be intact unless the dog was previously branded after being spayed.

A dog’s initial response to strangers can be helpful in evaluating a dog’s overall attitude towards humans and assess the likelihood of biting humans [2]. The observer had no prior contact with the dogs in this study, therefore, can be considered a stranger to the dogs. A modified “approach test” [2] was used to document the vocalization, dismissive, fearful and friendly behavior from the dog. The photographs and records from each observation were used to associate the identity of each dog and their co-occurrences.

Results from the previous study [32] were used to determine the transect route of observations. GPS coordinates were collected at the location of the visual observations to associate the location of the group of dogs with the previously defined areas. The observer also documented evidence of caretakers in the area such as a feeding bowl or leftover dog food. The mobile application, Epicollect5 [33,34] was used to facilitate the data collection and recording.

Observations were carried out on 16 different days across four months (September to November 2020). On average, 6–7 polygons were observed in one day. The average duration of one observation was 7 min and 31 s (range 1–28 min). Records of the observations along with the corresponding photos from different days were collated and examined to eliminate duplicate records of the same dog.

The present study was conducted during the COVID-19 pandemic, which meant the majority of classes were held online and students were off-campus. However, the faculty and staff were not required to work remotely. The amount of human activity, on campus, during the studied time should be similar to the amount of activity during summer and winter breaks.

### 2.3. Data Analysis

All the observation data were collected using Epicollect5, which was later imported and analyzed using R (Version 4.0.3) [35]. After the identity of each dog was verified, the data were transformed into a node list. The attributes (age, sex, castration status and behavior towards humans) were also included in the node list. An edge list of co-occurrences between dogs was created from the list of dogs encountered during each observation. Co-occurrence is defined as the presence of dogs in the same polygon within the same observation period. The node and edge lists were then utilized to create the one-mode (dog-to-dog) network. For the two-mode (dog-to-polygon) network, the edge list was created by relating the location of the dog to the polygon by which it was observed, using QGIS (Version 3.10.11) [36,37]. The node list of polygons included attributes regarding the possible food source and type of area (urban or rural). The polygon node list and dog node list were combined into the two-mode node list in order to create the two-mode network.

The network analysis software, UCINET (Version 6.720) [38] and Gephi (Version 0.9.2) [39] were used to create and visualize an overall dog-to-dog network (for the entire study duration) of all the dogs observed during the same observation period from the node and edge list described earlier. The grouping behavior of dogs is suspected to be influenced by the amount of human activity on campus and their feeder, which are only active during the weekdays. Therefore, two networks were created separately for dogs observed on the weekdays and weekends. In addition, the dog-to-polygon network was created using the previously described two-mode node and edge lists to describe the assumed association of the dog with the areas of interest.

Network statistics were computed for all one-mode networks, including network diameter, average path length and density. Components within the networks were identified and fragmentation of the network was calculated. Degree centrality and normalized degree were computed for each network. Statistical tests with permutation-based significances provided in UCINET were used to compare normalized degrees between different networks or groups within a network.

The main component is the largest connected group of nodes in the network. Normalized betweenness (nBetweenness) and normalized closeness (nCloseness) were computed from the main component of each network. The cut-points of the main component from the overall dog-to-dog network were identified using NetDraw (Version 2.175) [40]. Furthermore, the cut-points were removed and sub-groups were identified within the main component.

In addition, for each of the one-mode networks (overall, weekday and weekend), five hundred random networks of equal size and density were generated. The average path length (L) and average clustering coefficients (C) of the random networks of the same size and density were calculated and compared with the observed network to determine whether it had a small-world structure. Small-world networks have a higher clustering coefficient and lower average path length compared to the random networks. Furthermore, an alternative and simplified approach was also used to assess the small-world-ness (S) of a network as proposed by Humphries et al. [41,42] using the following formula:

S = (C_observed_/C_random_)/(L_observed_/L_random_) (1)

Networks that have S > 1 are defined as small-world networks.

Network statistics computed for the two-mode network include the density, transitivity, fragmentation and average path length. Two-mode centrality indices calculated include the average degree, nBetweenness and nCloseness. The two-mode degrees were further analyzed using statistical tests with permutation-based significances in UCINET.

A permutation-based simple linear regression model was used to determine the relationship between the normalized degree of dog nodes, calculated from the two-mode network and the nBetweenness, calculated from the main component of the overall dog-to-dog network. The model was fit using the normalized degree of dog nodes to predict the nBetweenness.

## 3. Results

### 3.1. Descriptive Analysis

A total of 261 dogs were recorded and identified from 113 observations during the study period at various locations on campus. Fifty-one observations were conducted during weekdays and 62 observations were conducted on the weekends. The observed areas extended into 42 polygons, of which 20 and 22 polygons were classified as urban and rural areas, respectively. One hundred and twelve dogs were observed only on weekdays, 80 were observed only on weekends and 69 were observed on both weekdays and weekends. The majority of dogs identified were adults (247/261) and only a few puppies were spotted (14/261). There were 101 female and 90 male dogs identified, 70 dogs could not be categorized for sex. Only 5% (5/102) of females were spayed and 11% (9/81) of males were neutered. The most exhibited behavior towards a human stranger during the first encounter was fear (131/261), followed by disregard (64/261), friendly behavior (52/261) and barking (14/261) (Table 1).

### 3.2. One-Mode Networks (Dog-to-Dog Networks)

**Figure 2:**
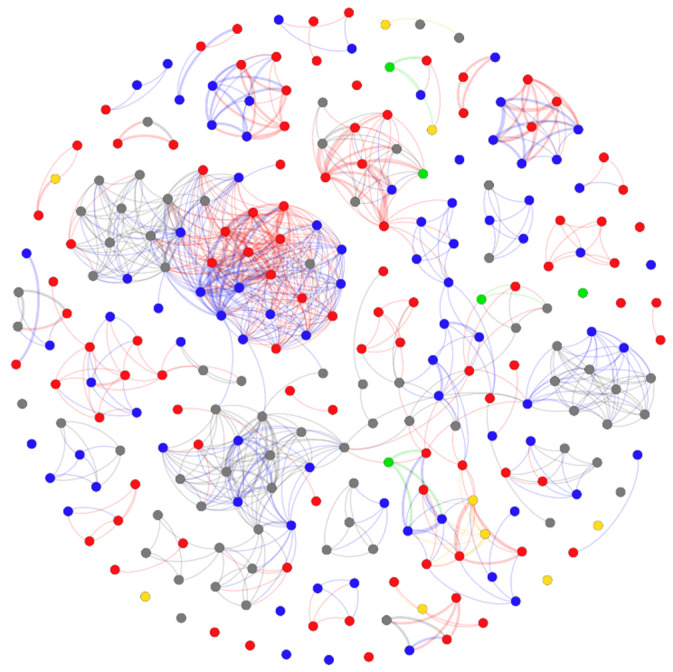
The overall dog-to-dog network graph, created in Gephi. The nodes are free-roaming dogs observed during study in Kasetsart University, Kamphaeng Saen, in 2020. The edges represent the co-occurrences of the dogs. The colors of the nodes represent the sex and castration status of each dog: male (blue), female (red), neutered male (yellow), spayed female (green) and unknown (grey).

The overall dog-to-dog network (Figure 2) contained a total of 261 nodes (dogs) and 1760 edges (co-occurrences). The average degree was 6.74 (range 0–29). Over fifty percent of all observed dogs each had degree ≤ 5 (150/261) and only 3.83% (10/261) had degrees over 20. Additional measures of centrality are displayed in Table 2. Fifty-four components were identified in the network. There were 20 isolated nodes. The largest three components contained 117 (45%), 13 (5%) and 8 (3%) dogs, respectively. The component ratio is calculated from the number of components minus one divided by the number of nodes minus one.

The overall dog-to-dog network had a high clustering coefficient (1.131) compared to the average clustering coefficient calculated from the 500 random networks (0.026). Furthermore, the S computed from the average path length and clustering coefficient was 25.148, which further confirmed that this network exhibited characteristics of a small-world network (S > 1).

**Figure 3:**
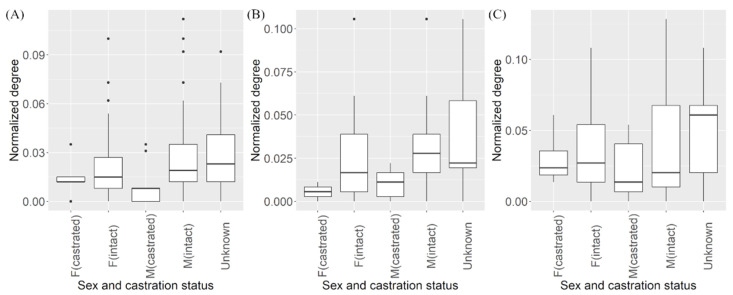
Plots of the normalized degrees stratified by the sex and castration status. Distribution of normalized degrees calculated from the (**A**) overall, (**B**) weekday and (**C**) weekend dog-to-dog networks observed in Kasetsart University, Kamphaeng Saen in 2020. The dots represent extreme values in the dataset.

Permutation-based ANOVA was performed to assess the difference between normalized degree distribution of studied dogs based on their sex and neuter status. However, no statistical difference was observed (*p*-value > 0.05, F = 1.849, df = 4) (Figure 3A) (Table S1). Cut-points within the largest component were generated in NetDraw. Removal of the cut-points in the main component created 10 smaller components and two isolates (Figure S1).

### 3.3. Comparison between Weekday-Weekend One-Mode Networks

Separate one-mode networks were created for dogs encountered during the weekdays and weekends to assess the effect of human activity on campus on the grouping behavior of dogs. The network of dogs observed on weekdays had 181 nodes and 1066 edges with an average degree of 5.89. The network was highly fragmented (97.4%), with a total of 50 components and had small-world topology. The largest component included 20 nodes or 11% of the total number of nodes. The clustering coefficient for the weekday network was 1.05 and the average path length was 1.08.

The network of dogs observed on the weekends had similar characteristics. The clustering coefficient was 1.05 and the average path length was 3.30, consistent with the small-world network topology. The weekend network had only 149 nodes and 902 edges with an average degree of 6.05. It was also highly fragmented (79.3%), having fewer components (33) compared to the weekday network. The largest component contained 66 nodes or 44% of the total number of nodes. The permutation-based t-test showed the normalized degree of dogs’ contacts during the weekends (average = 0.041) was significantly higher than on the weekdays (0.033) (*p*-value = 0.01, mean difference = 0.008) (Figure 3B,C) (Table S1).

### 3.4. Two-Mode Network (Dog-to-Polygon Network)

**Figure 4:**
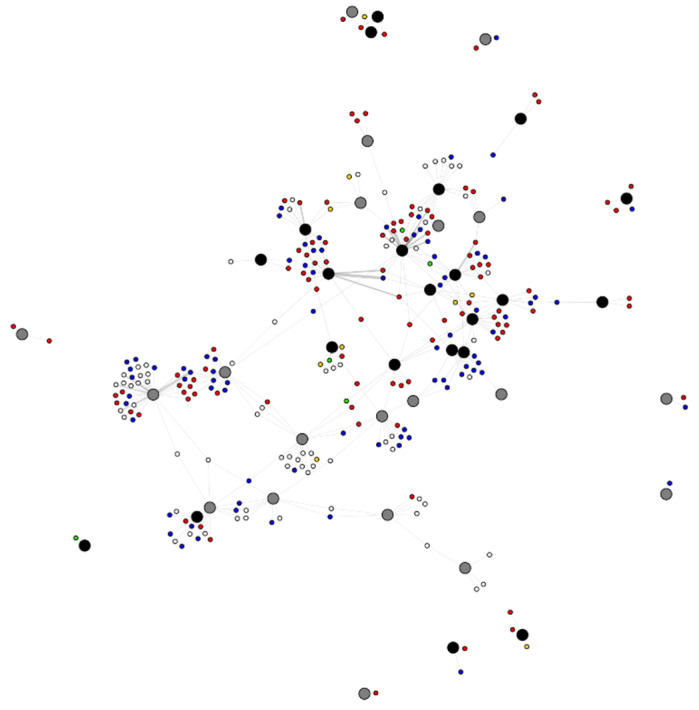
The two-mode dog-to-polygon network graph studied in Kasetsart University, Kamphaeng Saen in 2020, created in Gephi. The large circular nodes represent polygons. They were classified as urban (black) and rural (grey), according to buildings and human activity in the area. The small circular nodes represent dogs according to the sex and castration status: male (blue), female (red), neutered male (yellow), spayed female (green) and unknown (white).

**Figure 5:**
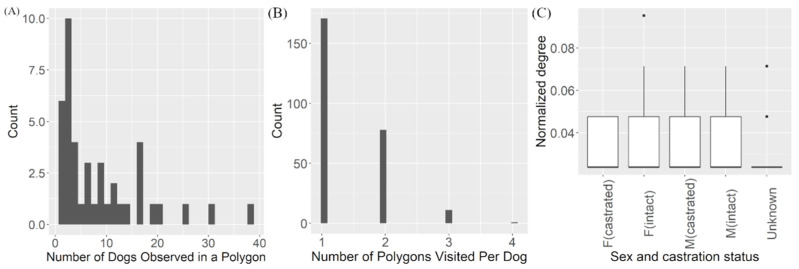
Histograms depicting the (**A**) degree of polygon nodes and (**B**) degree of dog nodes calculated from the two-mode network and the (**C**) box-plot of normalized degrees of dog nodes according to sex and castration status. The two-mode dog-to-polygon network measures included the degrees and normalized degrees of the two respective modes. Histograms depicting (**A**) the degree of polygon nodes (can be interpreted as the number of dogs observed in the same polygon (**B**) the degree of dog nodes (the number of polygons an individual dog was associated with) and (**C**) Box-plots showing the distribution of normalized degrees calculated from dog nodes was used to visualize the average number of polygons visited according to the sex and castration status. The dots on the plot represent extreme values.

A two-mode network (Figure 4) was created to visualize and quantify the overlapping area-use between dogs. The network had 303 nodes and 364 edges in total. The average degree of dog nodes, which indicates the number of polygons it appears in, was 1.40 (range 1–4). The average degree for the polygon, representing the total number of dogs observed in each polygon, was 8.67 (range 1–38). A summary of network measures from the dog-to-polygon network is displayed in Table 3. The normalized degrees of urban and rural area nodes (polygons) were compared using a permutation-based *t*-test (Table S1). However, no significant difference between the number of dogs associated with each area type was detected (*p*-value > 0.05) (Figure 5A).

A significant association was found between the normalized two-mode degree and nBetweenness of dog nodes in the main component (*p*-value = 0.01, F(46,1) = 7.102, R-square = 0.134) by using a permutation-based simple linear regression model (Table S1).

## 4. Discussion

The networks of free-roaming dogs in Kasetsart University, Kamphaeng Saen Campus, Thailand were created and characterized in this study. This is likely the first study in Thailand to establish contact patterns of community dogs inhabiting a shared space. The computed degree centrality from the overall network represented the contacts between each pair of dogs. Small-world characteristics were observed in the studied networks. In addition, we also identified potential cut-points in the network based on their betweenness, which can be applied to target interventions and formulate specific disease mitigation strategies. Dogs with high risk of disease spread can be assumed using the identified network measures such as high degree and high betweenness. For example, a dog with high risk of disease spread can be prioritized for vaccination or captured, to minimize the further spread within the connected network. Network modelling can also be used to predict the potential disease spread and evaluate the efficacy of the vaccination.

In a previous study of free-roaming dog networks in Torres Strait, Australia [27], three networks were created, one from each community. The networks were well connected. In two networks (out of three), all dogs were connected within a single component. The small-world structure was observed in all three networks. Similarly, the networks of owned free-roaming dogs in Chad, Guatemala, Indonesia and some sites in Uganda [28] were also well connected and displayed small-world characteristics. The observed dog-to-dog networks in our study also demonstrated a high clustering coefficient and short average path length, characteristics consistent with small-world topology. Although the average path length is longer when compared to the random networks, in smaller networks (200–3000 nodes), having a higher clustering coefficient is indicative of being a small-world network [42,43]. Furthermore, we also computed the network small-world-ness (S) value to confirm this finding. In small-world networks, an infectious disease can spread quickly within clusters and reach distant individuals within the network in a small number of steps [44,45].

The distribution of degree in the dog-to-dog network, including the weekday and weekend subsets, showed similar right-skewed distribution to the degree observed in the networks of dogs in previous studies [27,28]. These authors reported that dogs were generally associated with a few dogs for long durations while larger gatherings occur in shorter periods of time [27]. This is consistent with the findings in our study. No significant difference was observed when comparing the normalized degrees of dogs with different sex and castrations status in the dog-to-dog network and the dog-to-polygon network. Therefore, we could not conclude whether there was an influence of dog’s gender on clustering and diffused movement.

The lower degrees observed during the weekdays compared to the weekends could reflect the effect of external factors, such as the presence of people and automobile traffic, which affect group dispersal [46]. Conversely, considerably less human activity and traffic are seen on campus on the weekends, resulting in larger groups observed. This finding was initially thought to be due to the result of human-shy dogs appearing in the weekend groups. However, the proportion of the groups stratified by behavior showed the number of fearful dogs was equally high in both weekday and weekend networks (Figure S2). It is more likely that the dogs tend to gather in smaller groups in the presence of humans regardless of its’ familiarity with people. This has been previously discussed as a possible consequence of larger groups attracting unwanted attention [46]. Therefore, it is naturally more beneficial for the dogs to remain as a small group in the presence of humans. Another possible explanation could be related to the absence of a food source. The lack of a feeder or human refuse during the weekend could motivate the dogs to aggregate and roam further in search of food [47]. This was also observed during previous studies on campus [32]. Our finding of increased degree centrality in the absence of humans is in agreement with previous studies which observed the roaming activity of dogs to be associated with human activity [48]. Moreover, this association should be further investigated to see the extent of its’ effect and explore the possible uses in disease control planning. For example, there could be substantially larger gatherings during summer or winter breaks. Therefore, vaccination or capture-neuter-release programs may be planned accordingly.

Human activity has been shown to influence the gathering of dogs in a previous study in Ayutthaya, Thailand [49]. The networks for the dogs observed on weekends and weekdays were created to demonstrate the difference in dog-to-dog contacts occurring, as suspected to be influenced by human activity. Since the caretakers often feed dogs from Monday to Friday, we expected to see higher connectedness in the weekday network. However, the size of the network was the only difference observed between the weekday and weekend networks. Since, there was no evidence that the dogs observed during weekdays and weekends were distinct from one another and the overall, weekday and weekend dog-to-dog networks showed similar characteristics including high clustering, short average path length and high fragmentation, the understanding of disease transmission in this particular dog population should be based on the overall network.

Fragmentation of a network reflects the proportion of nodes that are not joined by direct or indirect edges. The observed dog-to-dog networks had high fragmentation, leading to a large number of components in the network. A component is defined as a group of nodes connected by at least one edge. The largest component was used to calculate the nBetweenness and nCloseness since all the nodes were not connected within the network. The largest component in the overall network had 117 connected nodes. For exploratory purposes, targeted removal of cut-points in the largest component could create up to 12 smaller components (including isolates). Roaming range was not explicitly measured and was beyond the scope of this study. In the study area, a dog could hypothetically roam across the entire campus area, although previous studies [32,50] and comments from feeders suggest that they are sedentary and have a high affinity to certain location. Because the spatial proximity enables them to spread disease, we included all dogs on campus, despite their affiliation with a community. Theoretically, identifying the dogs that act as the bridge between subcomponents (called “communities”) and severing the edges between subcomponents of dogs could decrease the risk of disease spread between clusters and, therefore, decrease the speed of disease spread in the event of an outbreak. In terms of rabies control, vaccinating the dog or capturing and restricting its’ movement has the potential to prevent the infection and stop the spread of disease. The ability to identify and prioritize high-risk dogs for intervention will be useful in developing targeted rabies control strategies.

Betweenness is measured as the number of shortest paths that pass through the node. Dogs with high betweenness would likely be the dogs that create edges between components and may have considerable influence on the network. In our study, the same group of dogs was found repeatedly in the same area. Therefore, it was also highly suggestive that the dogs with high betweenness roamed between multiple observed locations. In our study, the dog with edges to the most polygons (dog number 42) was identified as an intact female who exhibited friendly behavior to humans. There was no documented information on her reproductive status, for example, whether she was in estrus, which could have influenced her roaming patterns [27,48,51]. She was observed in four polygons (all categorized as urban) in total. During observations, this dog was observed with up to six other dogs and in total, she had contacts with 14 other dogs during the study. Dog number 42 was also included in the main component and her nBetweenness was high (14.89, while mean betweenness = 3.95). Dogs with high nBetweenness have great potential for disease spread. This is even more evident when roaming behavior (quantified by the number of polygon degrees computed from the two-mode network) is considered, as demonstrated in our study.

The degree calculated from the dog-to-polygon two-mode network in our study was highly suggestive of affinity of a dog to a place [47,52] as most of the studied dogs were repeatedly seen in the same polygon. Interestingly, there was no statistical difference in the number of dogs between urban and rural polygons. This suggests that other factors could likely play a more important role in determining the carrying capacity of dogs in each area. For instance, areas supervised by at least one human feeder could have more plentiful resources, sustaining and attracting a larger number of dogs. Further studies should be conducted in order to determine the important factors involved in the carrying capacity of free-roaming dogs in Thailand.

Previous studies [44,53] have demonstrated the benefits of using network models and contact network epidemiology to provide meaningful insights into the complex interaction between disease transmission and the contact patterns of free-roaming dogs. Estimating the speed and extent of the spread of a certain disease, such as rabies, and identifying a possible mitigation strategy specific to the network can result in developing practical control measures for the specific disease.

Considering the observational methods used in our study, the cross-sectional nature of the data collection only described a snapshot of potential contacts, rather than a continuous monitoring of dogs’ movements and contacts such as demonstrated in studies obtained using GPS tracking. Moreover, observational methods required the observer to be in close proximity with the dogs, which could have affected their behavior. However, the results produced from this study prove observational methods to be a simple and cost-effective alternative that can be applied in rural and resource-limited settings, where network data could be extremely valuable. Although the data used in this study were sufficient to create the networks of interest, a longitudinal study that can capture dog movements and their contacts continuously for the entire study period would likely provide additional valuable insights. Further validation of the cross-sectional design used in this study against concurrent longitudinal data will be useful in determining the similarities between networks generated from cross-sectional and longitudinal data.

The socio-ecology of free-roaming dogs is being increasingly studied in Thailand. In comparison, many common behavioral traits are shared with free-roaming dogs from other countries, such as affinity to a certain place with varying human familiarity [2,52]. The abundance of food provided by human feeders has created a distinct environment for the free-roaming dogs in Thailand. Previous studies have reported most of the dogs encountered appear to be in good body condition, very few dogs were emaciated [50,51,54] Furthermore, a study in Phitsanulok, Thailand, described a cluster of free-roaming dogs co-existing with a group of labor workers, similar to what we observed in this study [50]. In our study, we observed that most dogs on campus receive food from a caretaker. This is commonly observed in Thai communities. Moreover, two patterns of dog-caretaker associations that were previously observed in Prachuap Khiri Khan, Thailand [55] were also observed in this study. The dogs that are friendly and could be easily restrained by the caretaker and the dogs that do not tolerate any kind of human contact. These two distinct types of dogs are frequently observed in other rural communities in Thailand. Even though our study was based inside a university campus, we expect to see similar contact patterns and network characteristics in free-roaming dog populations with a caretaker associated with places with regular working hours.

## 5. Conclusions

This study described the network of community dogs on a university campus in Thailand. The free-roaming dog network described in this study had a small-world network characteristics with a right-skewed degree distribution, consistent with previously reported free-roaming dog networks in other countries. The high betweenness and high normalized degree measures were used in identifying dogs that had a higher likelihood of transmitting disease. The contact patterns of dogs on the campus were largely influenced by human activity. The weekend network showed that dogs tend to form larger groups during weekends, in the absence of humans. The two-mode dog-to-polygon network revealed that most dogs had an affinity to a place. The number of dogs observed in an area was not different between urban and rural areas likely because there were caretakers providing food in both types of areas. This study was conducted using a cross-sectional design; further studies should be conducted in order to determine the similarities of the networks created using a longitudinal study design. The network information acquired from this study can be further used in network modeling and designing targeted disease control programs.

## Figures and Tables

**Table 1:** Descriptive characteristics of free-roaming dogs observed in Kasetsart University, Kamphaeng Saen.

Characteristics of Dogs	Number of Dogs	Percentage
Age		
Adult	247	94.64
Juvenile	14	5.36
Sex		
Female	96	36.78
Male	81	31.03
Spayed females	5	1.92
Neutered males	9	3.45
Unknown	70	26.82
Behavior towards human during the first occurrence		
Bark	14	5.36
Dismissive	64	24.52
Fearful	131	50.19
Friendly	52	19.92
Total	261	100

**Table 2:** Network characteristics of the dog-to-dog networks observed in Kasetsart University, Kamphaeng Saen Campus.

Network Characteristics	Overall Dog-to-Dog Network	Weekday Dog-to-Dog Network	Weekend Dog-to-Dog Network
Total number of nodes	261	181	149
Total number of edges	1760	1066	902
Average Degree	6.743	5.890	6.054
Density	0.026	0.033	0.041
Average Distance (SD)	5.390 (3.358)	1.084 (0.324)	3.304 (1.601)
Diameter	14	3	7
nBetweenness (SD) ^1^	3.953 (9.692)	0 (0)	3.819 (10.249)
nCloseness (SD) ^1^	18.952 (3.834)	100 (0)	29.931 (5.347)
Small-world-ness (S)	25.148	142.168	28.706
Components	54	50	33
Component Ratio	0.204	0.272	0.216
Size of largest component	117	20	66
Fragmentation	0.792	0.974	0.793

^1^ The nBetweenness and nCloseness were calculated from the largest component.

**Table 3:** Network characteristics of the two-mode dog-to-polygon networks observed in Kasetsart University, Kamphaeng Saen Campus.

Network Characteristics	Value
Number of total nodes	303
Number of total edges	364
Density	0.033
Transitivity	0.698
Fragmentation	0.235
Average Distance	5.706
Diameter	14
Norm Distance	0.403
Radius	1
	Dog nodes
Number of dog nodes	261
nDegree ^1^	0.033
Average Degree (min–max) ^1^	1.395 (1–4)
Average nBetweenness ^1^	0.006
Average nCloseness ^1^	0.259
	Polygon nodes
Number of polygon nodes	42
nDegree ^1^	0.033
Average Degree (min–max) ^1^	8.667 (1–38)
Average nBetweenness ^1^	0.05
Average nCloseness ^1^	0.142

^1^ The normalized degree, average degree, nBetweenness and nCloseness were calculated for the respective modes.

## Data Availability

The data presented in this study are available in this article.

## Supplementary Materials

The following are available online at https://www.mdpi.com/article/10.3390/vetsci8120299/s1, Figure S1: Cut-points and subcomponents identified within the main component of the overall dog-to-dog network, Table S1: Summary of all permutation-based statistical analyses performed, Figure S2: Plots showing the number of dogs from the overall, weekday and weekend dog-to-dog networks stratified by behavioral response to an unfamiliar human.

## Appendix A

Assumed human activity, for each of the polygon, was ranked from low (<20 persons), moderate (20–100 persons) and high (over 100 persons), based on Kasetsart University’s report of personnel in 2019 [56] and observations during data collection. Human activities for areas not covered in the report, including cafeterias and lecture halls, were estimated from visual observations and categorized according to the criteria described above. A satellite photograph [57] of the campus was used to assess the occupancy of human-made structures within the polygon. According to a satellite image of the campus, polygons were ranked based on the proportion of area occupied by man-made structures within the polygon. It ranged from high (>0.5), moderate (0.25–0.5) and low (<0.25).
