# Supplementary material for: Analyses of Contact Networks of Community Dogs on a University Campus in Nakhon Pathom, Thailand
Source: Vet Sci. 2021 Nov 30;8(12):299. doi: 10.3390/vetsci8120299 (PMC8704209; doi:10.3390/vetsci8120299)
Supplement: Supplementary file 1 [file vetsci-08-00299-s001.zip › Figure S1.pdf]

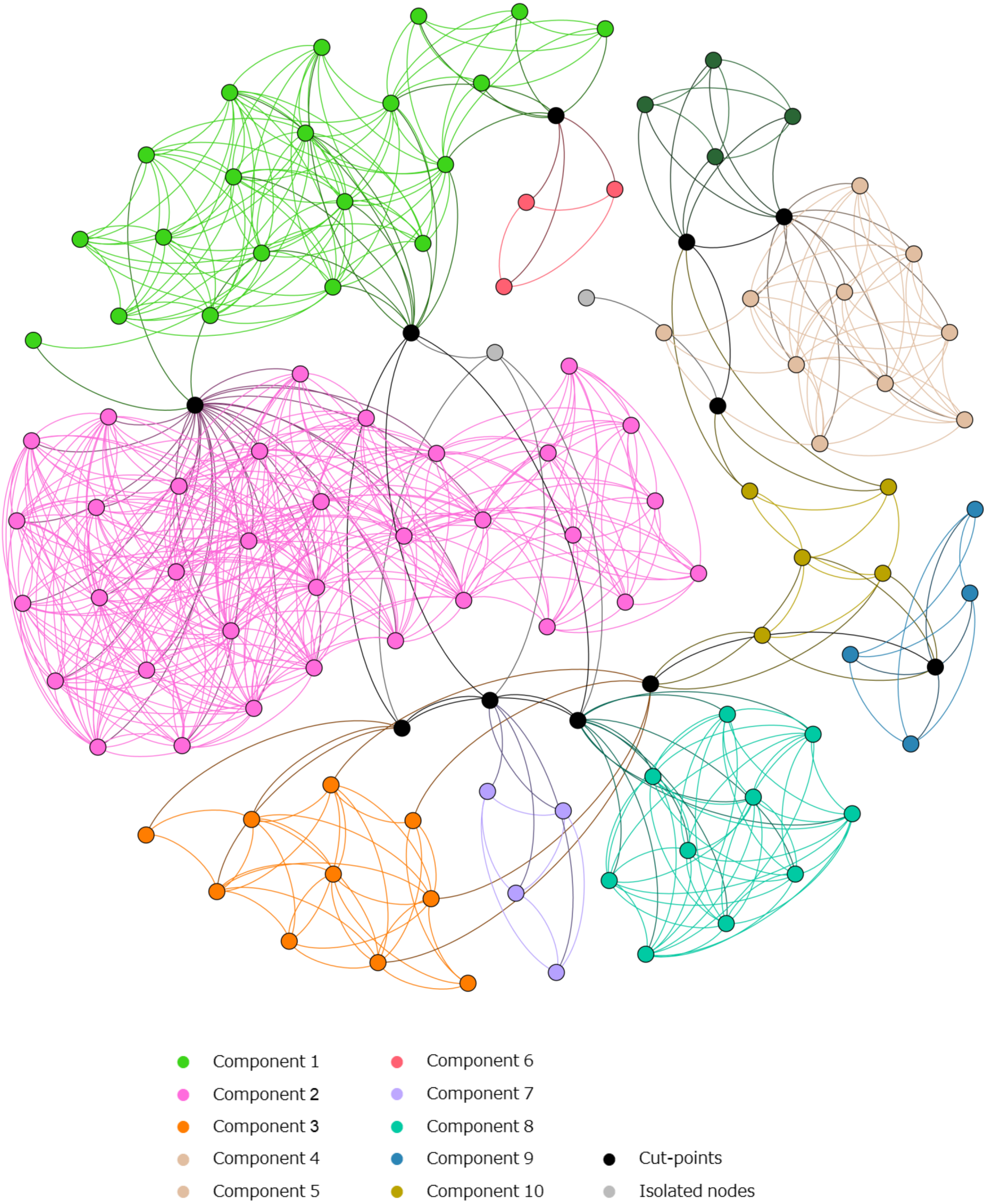
Component 1
Component 2
Component 3
Component 4
Component 5
Component 6
Component 7
Component 8
Component 9
Component 10
Cut-points
Isolated nodes
