# Supplementary material for: Analyses of Contact Networks of Community Dogs on a University Campus in Nakhon Pathom, Thailand
Source: Vet Sci. 2021 Nov 30;8(12):299. doi: 10.3390/vetsci8120299 (PMC8704209; doi:10.3390/vetsci8120299)
Supplement: Supplementary file 1 [file vetsci-08-00299-s001.zip › Table_S1.pdf]

Table S1

Statistical test	Dependent variable	Independent variable	Results
t test	Normalized degree (weekday and weekend dog-to-dog networks, respectively)	Weekday and weekend dog-to-dog networks	μ_1= 0.033, μ_2 = 0.041 p = 0.012*
t test	Normalized degree calculated from polygon nodes (dog-to-polygon network)	Urban and rural polygons	μ_1= 0.031, μ_2 = 0.035 p = 0.367
ANOVA	Normalized degree (overall dog-to-dog network)	Sex and castration status	F (4,256) = 1.849 p = 0.112
ANOVA	Normalized degree calculated from dog nodes (dog-to-polygon network)	Sex and castration status	F (4,256) = 1.616, p = 0.165
Linear regression	Normalized betweenness calculated from the main component (overall dog-to-dog network)	Degree calculated from dog nodes (dog-to-polygon network)	F (46,1) = 7.102 R-squared = 0.134 p = 0.012*

* Significant test/association
