# Supplementary figures and images for: Analyses of Contact Networks of Community Dogs on a University Campus in Nakhon Pathom, Thailand
Source: Vet Sci. 2021 Nov 30;8(12):299. doi: 10.3390/vetsci8120299 (PMC8704209; doi:10.3390/vetsci8120299)

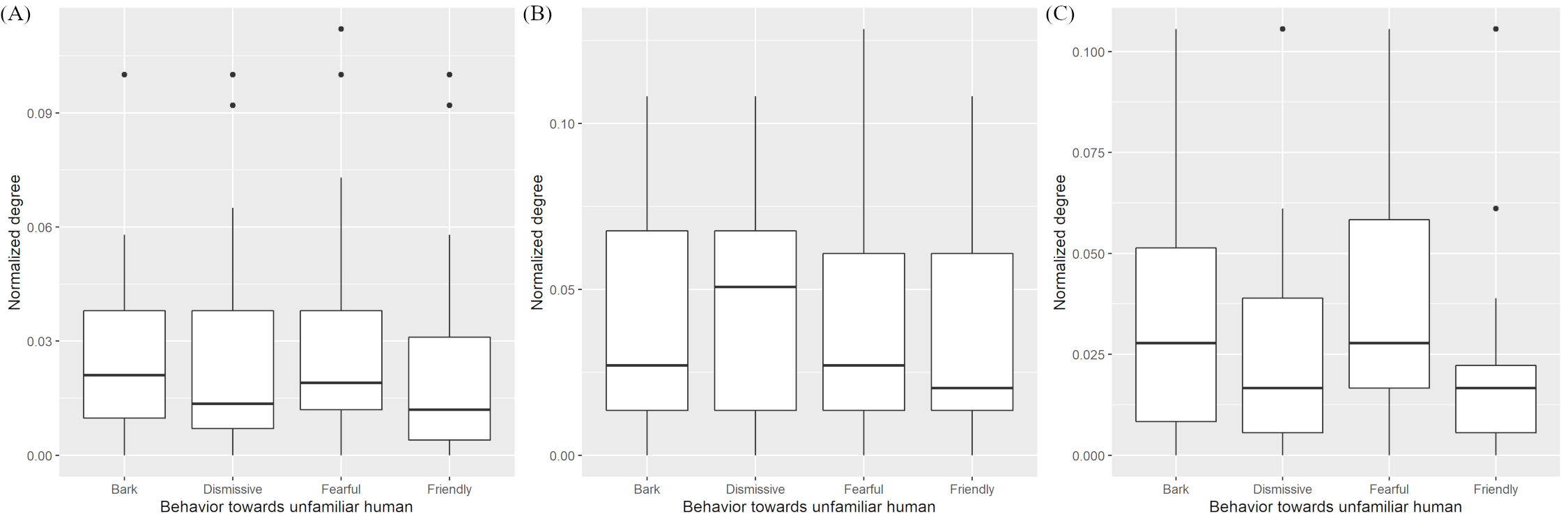

Supplement: Supplementary file 1 [file vetsci-08-00299-s001.zip › Figure S2.tiff]
